# Exploring the Role of Ad Hoc Grassroots Organizations Providing Humanitarian Aid on Lesvos, Greece

**DOI:** 10.1371/currents.dis.bd282cd90ade7d4eb63b6bbdb1904d10

**Published:** 2016-11-17

**Authors:** George Tjensvoll Kitching, Hanne J. Haavik, Birgit J. Tandstad, Muhammad Zaman, Elisabeth Darj

**Affiliations:** Department of Public Health and General Practice, NTNU, Norwegian University of Science and Technology, Trondheim, Norway; Department of Public Health and General Practice, NTNU, Norwegian University of Science and Technology, Trondheim, Norway; Department of Public Health and General Practice, NTNU, Norwegian University of Science and Technology, Trondheim, Norway; Department of Biomedical Engineering, Boston University, Boston, USA; Department of Public Health and General Practice, NTNU, Norwegian University of Sciences and Technology, Trondheim, Norway; Department of Obstetrics and Gynecology, St Olavs Hospital, Trondheim, Norway; Department of Women’s and Children’s Health, Uppsala University, Uppsala, Sweden

**Keywords:** community networks, disasters, emergencies, refugees, surge capacity

## Abstract

Introduction: Syrian refugees displaced into Turkey have attempted high-risk sea migrations to reach safer destinations in Europe, most often initially arriving on the Greek island of Lesvos. These refugees were often in need of basic humanitarian assistance that has been provided in part by a new category of ad hoc grassroots organizations (AHGOs). The aim of this study was to understand the internal and external operations of these AHGOs and their role on Lesvos.

Methods: The experiences of AHGOs were investigated through a qualitative research design utilizing semi-structured interviews with organization leaders and spokespersons. AHGOs identified through media and social media sources as new Lesvos-specific organizations were purposively invited to complete an interview over phone, Skype or email. Data analysis of the transcribed interviews was performed by Systematic Text Condensation.

Results: Forty-one organizations were contacted and 13 interviews were conducted. Most organizations were formed in autumn 2015 responding to the greater influx of refugees and migrants at that time and reported an absence of professional humanitarian agencies providing aid on Lesvos. Three categories emerged from the material. Features of organizations; Features of volunteers and; Evolution of AHGOs. The organizations perceived themselves capable of evaluating needs, mobilizing resources, funding and providing quick response. The volunteers came with limited humanitarian experience and from a wide variety of nationalities and professional backgrounds, and the organizations developed while on Lesvos.

Discussion: Knowledge from our findings of AHGOs response to this complex disaster on Lesvos could be utilized in future catastrophes. We conclude that AHGOs may prove effective at providing humanitarian aid in a surge response when international non-governmental organizations are unable to respond quickly. In future complex disasters AHGOs should be recognized as new humanitarian actors and conditions should be made favourable for their operations.

## Introduction

The ongoing conflict in Syria and the surrounding region has led to the displacement of people within and out of Syria, into neighbouring countries within the region^1^. The total number of Syrians displaced out of Syria was estimated to have reached 4.3 million by the end of 2015^1^. The United Nations (UN) has repeatedly requested other countries outside of the region to accept vulnerable refugees to ease the pressure on Syria’s neighbouring countries^2^.

Refugees in the neighbouring countries often attempt high-risk sea migrations to reach safer destinations in Europe. One major route involves crossing the Mediterranean Sea from Turkey to the archipelago of Greece in small over crowded vessels organized by smuggler groups^2^. In October 2015 was the peak period for crossings, with 135,053 arrivals to Lesvos, the most common point of arrival^3^. In the period before 20 March 2016 refugees and migrants arriving to Lesvos were registered and began a migration to other destinations in Europe. Hereafter a political agreement was implemented between the European Union (EU) and Turkey, which limited the movement of prior arrivals in Greece and secured deportation of new arrivals^4^.

The refugees and migrants arriving by boat are in need of basic humanitarian assistance^2^^,^ 5. Humanitarian aid organizations, both governmental and non-governmental, have worked to provide this basic assistance at many points on the path from the landing beaches. Media (grey sources) have indicated some of the organizations providing humanitarian aid are specifically focused to tackle this particular disaster and draw from a different volunteer base, than typical Emergency Medical Teams, as classified by the World Health Organizations (WHO)^5^^,^ 6 ^,^ 7 . In our study these organizations are called ad hoc, not existing before this disaster, and grassroots, organized by collaborations and partnerships between non-professional individuals. Limited information is available regarding the full picture of ad hoc grassroots organizations (AHGOs) active in Greece^5^. AHGOs have provided humanitarian relief to past disasters, such as the Earthquake in Haiti in 2010 and Hurricane Sandy in 2012^8^. While AHGOs continue to remain active on Lesvos following the EU-Turkey agreement, this report seeks to identify and characterize the emergent AHGO disaster response present in Greece focusing on the period prior to the major changes resulting from the agreement.

In this study we assign the term refugees and migrants to all peoples making the sea-crossing from Turkey to Lesvos in accordance with UN definition^9^^,^ 10. Using refugees and migrants attempts to capture the entirety of arrivals, simultaneously acknowledging that most people arriving on Lesvos are fleeing the regional instability resulting from the Syrian conflict, but also that not all migrants may qualify as refugees. The majority of interview respondents utilized the term refugee alone. The term International Non-Governmental Organization (INGO) refers here to all non-ad hoc grassroots, but international professional humanitarian non-governmental organizations active on Lesvos. However, acknowledging the diversity of organizations within this definition, we do not believe further refining this definition adds value to this report that aims to characterize the AHGOs response on Lesvos. The aim of the study was to investigate internal and external operations of AHGOs and to explore their perceptions and experiences of providing humanitarian aid during the crisis on Lesvos, Greece.

## Methods

**Study design**

A qualitative method with semi-structured interviews was chosen in order to explore the broad range of experiences of AHGOs on Lesvos, and not to evaluate effectiveness. Specific objectives were to explore perceptions of the AHGOs internal structuring, funding, volunteer base characteristics, coordination with others and specific aid services provided to the refugees and migrants, as well as to the volunteers.

**Study participants**

AHGOs were identified initially through searches on Google and social media sites, Facebook and Twitter, using combinations of keywords including: “Refugee”; “Syria”; “Asylum-seeker”; “Les(v)(b)os”; “Greece”; “Aid”; “Relief”; “Rescue”; “Humanitarian”. AHGOs identified through Internet search were also corroborated with information from the UNHCR Lesvos Contact List.

Participants were purposively invited through email, for voluntary contribution, by GTK. The target group was senior representatives within the AHGOs in order to gain insight into the inner workings of the organizations. Inclusion criteria were AHGOs, founded with the aim to relieve suffering on Lesvos and have or have had active volunteers providing aid on Lesvos. Organizations without active volunteers on Lesvos, or as being formed prior to this crisis in response to a different disaster, were excluded from the sample.

**Study setting**

Areas of interest for AHGOs included the towns of Molyvos, Eftalou and fishing village of Skala Sykaminias on the North coast and campsites of Kara Tepe, Pikpa and Moria in the Southern part of the island, close to the capital Mitilini. Moria was identified as the site of a registration camp operated by the European Agency for the Management of Operational Cooperation at the External borders of the Member States of the European Union (Frontex), and for a period of time in partnership with the UNHCR, Danish Refugee Council.

**Data collection**

Prior to the interviews, an interview guide was constructed with some specific questions regarding the characterization of the organizations and topics with open-ended questions to discuss regarding their operations and experiences providing aid on Lesvos (Appendix A). Interview guide questions were formulated through discussion and consensus of the authors. All interviews were conducted in English, tape recorded with the permission of the participants, and transcribed verbatim from the audio-recordings. Identifying features within the interview were removed to ensure anonymity.

Semi-structured interviews were conducted over Skype, Whatsapp call, phone call and for one interview, over email. Participants were asked as the last question within their interview, to identify other relevant organizations that could be added to the sample.

**Data analysis**

Data analysis of the transcribed interviews was performed by Systematic Text Condensation^11^. Mindjet manager software was used to sort, code and analyze extracted units of meaning. GTK, HJH and BJT worked to transcribe the interviews and to develop multiple levels of coding. They met several times to discuss the results and develop final subcategories and categories, which were further discussed with the international team of senior researchers. All authors had access to the material. Study participants were not contacted for comment or validation of the study findings.

**Ethics statement**

The study has been assessed by the Regional Committee for Medical and Health Research Ethics (REC) of central Norway. In the evaluation by REC of central Norway, no ethical permission was found to be required, as no health information, test results or biological samples were collected, only anonymized verbal interviews. Informed verbal consent was obtained from all participants prior to conducting interviews. Participants were informed about the aim of the study, asked for voluntary participation and were informed that they could withdraw from the interviews at any time, without giving any reason.

## Findings

We initially identified and contacted 41 AHGOs, and conducted 13 interviews in a six-week period from 25 January 2016 and 3 March 2016. Interviews lasted on average 50 minutes. One organization that provided an interview was subsequently excluded from the results because it was identified that, despite being a grassroots organization, it was not ad hoc as it had initially formed in 2005 and had been involved in other humanitarian activities since. Through the interviews several other AHGOs were identified beyond the initial that was found, and it was indicated that there were over 80 organizations, including INGOs, active on Lesvos. After 12 interviews, we decided the material was saturated as no new expressions evolved.

AHGOs interviewed originate from diverse nations including two from England, three from Greece, two from Holland, one from Norway, one from Palestine, two from Sweden and one from the United States of America.

Most groups and individuals that were later founded as an AHGO began providing humanitarian aid on Lesvos, before they went through the process of becoming an official organization or registering as such. The earliest surveyed began working in November 2014 and the latest surveyed in November 2015. Most of them started in September and October 2015 and were officially recognized a month later.

Trying to get an estimate of total volunteers, gave a mix of responses, such as total estimates, weekly rates of volunteers, and specific time period estimates. Collating this information gave estimates of 2260-4240 volunteers in the period of November 2014 to February 2016.

Three categories emerged during the qualitative analysis; Features of AHGOs; Features of the volunteer base for AHGOs, and; Evolution of AHGOs.

**Table 1:** **Table 1**. Categories and sub-categories for experiences of AHGOs active on Lesvos

Categories	Sub-categories
Features of AHGOS	Services provided to refugees and migrants
	Services provided to volunteers
	Internal structures of AHGOs
	Funding of organizations
	Coordination with others
Features of the volunteer base for AHGOs	Diversity of volunteers
	Recruitment
	Length of stay
Evolution of AHGOs	Development of AHGOs
	External conditions favouring AHGOs
	Ethical challenges perceived by AHGOs

**Features of AHGOs**

*Services provided to refugees and migrants*

AHGOs provided a diversity of emergency humanitarian relief services to refugees and migrants arriving on Lesvos and most organizations were involved in providing multiple services. Many boats arrived on beaches along the coast and organizations worked to receive boats, check health status of the arrivals and provide food and dry clothes. The Greek coastguard transferred people from arriving boats to the harbor of Molyvos and to AHGOs reception camps. A critical need for patrolling cars was acknowledged. Organizations stressed the importance of ensuring that medical personnel examined refugees and migrants and that families remained united. Some organizations had specialized teams of volunteers, pre-qualified to provide search and rescue services:

*“We recruit wilderness trained people… Lifeguards, paramedics, anyone who has wilderness training. Anyone who is able to operate in a remote environment cause this place we work at is like an hour away from anything where they could get real help. So they all bring the training with them. We provide the organization, the kit, the procedures, and the protocol of how to operate in that environment. Their personal skills are something they bring with them.” (Interview 1)*

In reception camps refugees and migrants could shelter, before traveling to the registration camp at Moria. Medical clinics were hosted where volunteers with medical and first aid qualifications assisted refugees and migrants. Storage warehouses existed to receive donations of goods and non-food items (NFI), such as clothing, and organizations sorted and distributed supplies to different locations around the island.

*“Clothes, for example is the biggest thing on Lesvos. There is both too many clothes and not enough of the right clothes on the whole island, but there is an interesting system of warehouses and Whatsapp groups to pull resources from different volunteer warehouses that somehow functions.” (Interview 1)*

Targeted campaigns were held in organizations home countries to accumulate particular NFI supplies and were perceived as successful both at home, in raising awareness, and also with the refugees and migrants.

Food was provided to refugees and migrants, either produced and distributed by the AHGO volunteers, or produced by a catering company on the island and distributed by volunteers. Food distribution was often coordinated between multiple organizations. Further the refugees and migrants were indirectly aided by AHGOs providing logistical support to other organizations, transporting donated items from warehouses and pick up points to areas of need based upon their requests.

Translation services for Arabic, Farsi and Dari languages were provided on the beaches, in support of other organizations and to the Greek police. Information was also provided to refugees and migrants regarding their continued journey and funeral arrangements for victims of sea-crossings. Organizations provided psychosocial support and guidance, including referral to specific clinics and services in destination countries, online chat support via Skype and Whatsapp, and designated safe spaces for vulnerable refugees and migrants including children.

The accumulation of life-vests and rubber boats discarded by the arriving refugees and migrants was identified as an environmental issue and organizations were engaged in beach cleanup activities.

*“…there is a great environmental problem, there are thousands of tons of life-vest and there are many boats. You can’t recycle, you can’t reuse them. This is a environmental bomb.” (Interview 3)*

Organizations considered ways to ‘up-cycle’ the discarded life-vests into products worth more than the discarded materials.

*Services provided to volunteers*

New volunteers received orientation training that varied between organizations. Orientations included learning work expectations, how the organization functions, the different roles of responsibility within the organizations, discussions about how to work effectively as volunteers providing humanitarian aid to refugees and migrants, and acted as a formal registration point within organizations.

The provision of task specific training varied among the AHGOs. They reported that volunteers brought their own qualifications for first aid and medical tasks. The simplicity of many of the other tasks such as warehouse sorting and distribution were considered not to require formal training beyond supervised learning through experience. Occasionally there were opportunities to send longer-term volunteers to do training provided by other organizations including training in psychological first aid, protection training, or first aid and cardiopulmonary resuscitation (CPR) Organizations that provided task specific training specified psychosocial training provided to volunteers in their home country, before they went to Lesvos, and significant training in the use of a secure online dispatch system for coordinating logistical support between organizations.

The ability to provide volunteers with accommodation and food varied. Volunteers could be completely responsible for their individual accommodation and food or organizations providing meal services designed to ensure that volunteers maintained their health:

*“…many people starting in the beginning, they totally forgot themselves, just ate when they got home, after so many hours just running on adrenaline and losing many kilos…” (Interview 5)*

The crisis put emotional strain on volunteers, including engaging in long hours, and ability to provide psychological support to volunteers ranged from a prescribed debrief service when volunteers returned home, to free access to a Red Cross psychological first aid provider and psychologist, or limited support when volunteers were on Lesvos, such as social debrief meetings. One way of support was to remain “in touch” with volunteers when they returned home and in some cases referral of volunteers to counseling within their home countries. AHGOs were aware that volunteers have difficult readjusting to their return home and plan return trips:

*“Once you’ve been in the real core of helping and dealing with situations, your whole life and perspective changes, it will never be the same again…. many of them cannot rewire to what they were doing before. I don’t know, it’s just like some connection gets lost with everything you did before, and you start to question everything. You start to question your own values, your life, it’s like a very life changing thing what happens to many people.” (Interview 5)*

*Internal structures of AHGOs*

AHGOs identified challenges, such as that few individuals within their organizations have any experience in humanitarian aid organizations before.

*“We’re now working on all kinds of official background documents, like codes of conduct and policies for the organization, that we of course need to have. Everything is a learning process because actually no one in the organization has ever run a foundation before… So we’re figuring out the wheel in a situation that actually barely anyone has experienced ever before.” (Interview 6)*

Leadership roles were identified to be past on within organizations also from the founders who could no longer stay or were moving on to other projects. Positions of differentiated responsibility were created as a result of identifying particular organizational needs in the moment. AHGOs had assorted internal structures, positions with differentiated responsibilities, and relied on short decision making chains. There were organization structures identified as non-hierarchical or flat, and those organized in different levels. Directors or founders were principle decision-makers, but were not necessarily consistently present on Lesvos and in some cases were responsible to an external board of directors or administrative team. They were often supported by a mix of coordinators with specific responsibilities such as coordinating volunteers, setting up work schedules, book keeping, keeping track of items, managing storage warehouses, managing external communications, managing medical units, managing reception sites, and logistics for a request-based transport services. The specific responsibilities of coordinators were dependent on the specific activities of the organization. The most complex structure reported included three levels of responsibility between the principle decision-maker and the general volunteer position. Responsibilities held were complex, as described for the case of logisticians remotely managing a supply distribution network between organizations:

*“So you have to figure out where your driver is, where the warehouses are, who has what, what the urgency levels are on each request, and put together a routing that makes the most sense. And then part therapist because we are, you know, operating in an emergency zone. Tensions are high, the volunteers on the island are often running on fumes.” (Interview 8)*

Long-term volunteers, active for at least one month, were also seen as providing a leadership role to volunteers who were active for shorter periods:

*“…we have long term volunteers who take up leadership roles, and they basically make sure there is a pass over of knowledge and everything we need to run smoothly.”(Interview 6)*

Communication of information between those in leadership roles and general volunteers was conducted in many different ways including in person meetings, phone calls and text messages. Whatsapp, a cellphone based messaging platform, and Slack, a web-based organizational platform, were identified by organizations as important communication channels, however Internet and cell service were identified to be unreliable.

*Funding of organizations*

Private donations of financial support accounted for almost all of the funding used by organizations active on Lesvos. It was identified that in autumn 2015 active volunteers provided the majority of donations and it created a certain feeling of goodwill. This changed, however, as organizations became more structured and opened bank accounts allowing organizations to receive funding directly from individuals who are not active volunteers.

*“And it’s lost the sort of, the camaraderie of everyone, throwing all their own personal money and their own time into something just to save lives because before there was very little money in the organizations because there were no, nowhere to donate. Everyone was using their own personal money to go out and buy food for refugees, to rent vehicles, and do stuff like this.” (Interview 1)*

Donors were now identified to be individuals and small groups from all around the world, though organizations that were registered in one country reported receiving most donations from that country. Organizations reported that financial donations were often more useful than donations of goods, allowing them to make purchases of food, clothes and materials locally on Lesvos based on needs in the moment. It was identified that volunteers conducted fundraising campaigns both to support their trips and raise money to donate to organizations.

AHGOs reported receiving donations of supplies and volunteers brought medical equipment based upon lists of needed medical supplies maintained by organizations providing medical support. Organizations identified corporate sponsors that provided funding or support through free shipping services of NFI items from communities across Europe to Lesvos. There was limited financial support from the local government or UNHCR.

The organizations defined themselves as non-governmental organizations, charities or in some cases social enterprises allowing them to use alternative fundraising methods. Social enterprises spoke of trying to involve local Greeks to create jobs on the island.

*Coordination with others*

The UNHCR hosted weekly “cluster-like meetings” which focused on specific clusters such as: protection, NFI, food, wash, shelter, and health. General coordination meetings with the UNHCR and local government, focused on new information, updates on policy implementation, addressing gaps in service provision, and developing a standardized method for receiving boats, including preparing for a mass casualty incident.

For some, cooperation between organizations ensured that services such as food preparation and distribution were covered every day, coordination through logistical services, communication on boat sightings and determined responsibility for ‘zones’ of coastline. Storage warehouses were also coordinated between organizations to ensure distribution of resources to areas of need. Communication between organizations was conducted through Whatsapp, phone calls, VHF radio along the coastline, and also in person at daily meals provided to organizations at the Better Days for Moria camp. Organizations also reported that they refused to cooperate with others unless necessary:

*“As a self organized, non governmental or official group, we are against the way NGO's and state are acting on the situation. However the need to provide to the refugees arriving the best possible treatment, forces the situation to cooperate in the time of need, for the good of these people[refugees and migrants].” (Interview 10)*

AHGOs reported mixed support from the local community, locals were included alongside international volunteers, but tension between locals and volunteers was also identified.

*“… The local people … feel… they can cope with the refugees arriving, but the volunteers are a second invasion and people here are starting to feel real fear for their future because of the economic impact, I guess that their currently having, loss of tourism.” (Interview 12)*

Local support extended in some cases to the local authorities, contact with the mayor, good relations with the Greek police and with the Greek coastguard.

**Features of the volunteer base for AHGOs**

*Diversity of volunteers*

There was a large diversity in the volunteers supporting these AHGOs. Volunteers came from countries around the world, with a large portion from European and North American countries.

*“…all together it’s just like it’s your neighbourhood, going to rescue refugees on Lesvos. All different backgrounds, different age, men and women…” (Interview 4)*

A variety of skill sets and employment history were provided by volunteers and limited experience providing humanitarian relief was widespread. At times searching for volunteers were focusing on specific skill sets such as engineers, search and rescue skills, medical and first aid skills or those with psychosocial support skills.

*Recruitment*

Initially over the summer of 2015 organizations providing humanitarian aid were supported by tourists. This volunteer demographic was acknowledged to change towards the end of the summer and into the autumn, as tourist season ended and international volunteers began to arrive expressly to provide humanitarian aid. Local Greeks were reported to have provided assistance throughout. Since the autumn organizations have had a mix of volunteers who either made prior contact or arrived independently and joined once on Lesvos. Volunteers who contacted organizations before coming, communicated through Facebook or organization websites and those who joined were assessed by organizations as to the length of their stay, and the particular needs of the organization in the moment.

*Length of Stay*

The period of time volunteers were active on Lesvos mirrored the diversity in the volunteers. While local volunteers remained consistently engaged, international volunteers were active from a few days up to over 8 months. Within this range organizations distinguished between short-term volunteers, those active between one and two weeks, and long-term volunteers, those active for over one month. Certain organizations implemented minimum time commitment requirements, such as a two week commitment, and referred volunteers who could only commit less to other organizations. Volunteers also returned for multiple periods and remained engaged once they return home. Visa restrictions were cited as a limiting factor in how long certain international volunteers could stay in Greece while others indicated length of stay was based around volunteers available work holidays.

**Evolution of AHGOs**

*Development of AHGOs*

AHGOs identified the desire to help to be a driving reason for many volunteers arriving on Lesvos to provide aid. This was coupled with shock and disappointment from the perceived lack of action from the UN and INGOs. AHGOs stressed the fluidity of the situation and the continual learning process. It was identified that part of the impetus to create organizations and formalized structures to support humanitarian aid provision was the existence, initially in the summer of 2015 and as the crisis intensified, of bad humanitarian practices.

*“…when summer was here there were an overwhelming number of boats and there was just a need to do whatever you could. But there was still a lot of bad practice. Families were split up because literally people [volunteers] just wanted to come and grab babies off boats… Families literally got separated. Lots of them, sometimes for hours, sometimes for longer. People [refugees and migrants] were not treated right for hypothermia, because they [volunteers] were too focused on handing out sandwiches instead of taking them to the clinic and getting off the beach.” (Interview 1)*

The process of becoming more formalized was identified as a learning process and that organizations were trying to develop best practice while staying true to their open grassroots beginning. Formalization was identified as one way to inhibit ‘cowboy’ groups from returning and engaging in bad humanitarian practices. Creating organizations and the process of formalization also extended to the need to cooperate between organizations.

*“And we’ve also learned that this work is very difficult. It requires a lot of cooperation with other NGOs, other organizations, you know the authorities, not something you can do on your own. Very much a team effort. To help one person you may need to be in touch with five or six different organizations or people. It requires a lot of cooperation with others.” (Interview 7)*

This process of formalization was partially attributed to external pressure and AHGOs developed unique strategies to speed registration processes including identifying that it was faster to become a social enterprise than to register as an NGO, and creating an umbrella organization with a general mission statement of supporting displaced people to ensure fast approval within their home country.

AHGOs were sensitive to identifying the needs of the refugees and migrants and the gaps in service. This sensitivity manifested in organizations that sought to either expand their operations both on Lesvos and in other locations or were in the process of contracting and ending particular services on Lesvos. Organizations visited other Greek islands, Turkey, Serbia, Macedonia and the Greek-Macedonian border to establish connections to other organizations and assess needs. Ending particular services was also considered because organizations did not want INGOs to become dependent on their efforts.

*External conditions favouring AHGOs*

AHGOs perceived that INGOs and the UNHCR were not providing significant aid. They reported that these INGOs were late in responding to the crisis and blamed the chaos experienced in October and November on their lack of action. Their initial absence was identified to be as a result of political pressure on Greece and their inability to respond quickly.

*“The reasons the whole response on Lesvos has sprung up the way it have, is because the majors [INGOs] weren’t there. You know, there is so much political pressure on Greece for not assisting in any way, on any of this. So yeah, the majors that you would expect to step in just were not operational on the island. That is why volunteers, individuals and small agencies came in from all around the world to fill that hole.” (Interview 8)*

Efforts to become a more formal organization were tied to an increasing presence of INGOs and the UNHCR. AHGOs identified their role in catalyzing INGOs and reflected that their own speed of action was an asset.

*“…they [INGOs] weren’t here and the only people who were saving lives before were the cowboy groups that we’re trying… at not being like them, but the reality was back then, the only group that could move fast enough were ordinary people with money and a lot of passion… How do we maintain the professionalism and the ability to move quick if something happens?” (Interview 1)*

Organizations reflected that the response from AHGOs on Lesvos raised questions for the field of humanitarian response.

*“Our perceptions about what is required and, you know, what we have to have in place to successfully run emergency response. It’s all put to question when you see what this little ragtag band [AHGOs]… has managed to pull off.” (Interview 8)*

*Ethical challenges perceived by AHGOs*

One aspect of the evolution and development of these organizations was the confrontation with ethical issues within the provision of humanitarian aid. AHGOs shared the sentiment:

*“We do not make distinctions between legal and illegal refugees, or the different countries they are coming from. The need to seek a better life is a right for everyone, as long as the conditions they are living in do not allow them to exist in dignity and safety.” (Interview 10)*

Organizations discussed the implementation of policies and increased control by the local authorities in the context of their cooperation efforts, especially at the registration camp at Moria, and responded in different ways.

*“…well we expect that soon they’re [Frontex] also going to start detaining people or detaining and deporting people back. Ya. Then by working inside the camp you’re also facilitating a system that you don’t necessarily support. We’ve always tried not to be a political organization but slowly we’re more and more forced to make a political stance…” (Interview 6)*

An ethical dilemma was raised as working in partnerships with the local authorities, INGOs and UNHCR could affect their ability to maintain their own values:

*“…the more organized things get on the island, the more the authorities, you know, take over certain parts which they should. I mean, the more we learn that, it’s very limiting to be in this line of work… the main challenge would be how can we still go through with what we want, to be able to help, and follow different types of rules and still maintain our values, what we believe in.” (Interview 7)*

In the face of increasing government control organizations also condemned cooperation and refused to support the actions of INGOs or the local authorities.

The registration process developed by the Greek government for individual volunteer-level and organizational-level registration was identified as beneficial to ensuring that organizations engaged in good humanitarian practices, or condemned for its subjectivity and for potentially contravening International Human Rights Law and European Law.

*“I think that it’s not a massive conspiracy to undermine peoples rights, I think it’s a rushed through thing because the government feels the need to take some control of this group of people [volunteers] who’ve come here. But in reality of what’s on the paper is very invasive, it’s alluding to illegal kind of consequences and it might. What scares me is that people fail to act in a situation where there is human need because they are afraid of unknown consequences then refugees don’t get the service that they have been getting…” (Interview 12)*

A perception was that volunteers faced personal moral dilemmas in response to this shifting regulatory environment. Furthermore a specific ethical issue was brought up, the inappropriate use of photos as exploitative of refugees and migrants in vulnerable situations.

*“In Greek law, it is illegal to take photographs of kids…people in particular need…without their consent. Even when a kid gives you permission, you need to explain the kids what you are going to do with it. It’s not allowed and it’s illegal to put refugees on Facebook or social media…99% of NGOs, volunteers are unaware of what they are doing…So there is a huge moral, how do you call this, contradiction to everything we are seeing on a daily basis.” (Interview 5)*

## Discussions

Our study characterizes the role AHGOs in providing humanitarian aid to refugees and migrants on Lesvos. Senior representatives of AHGOs active on Lesvos provided information regarding the internal and external processes of their organizations. Respondents also spoke to the development of AHGOs and the external pressures that affect their operations.

*Surge Capacity*

A review conducted in 2007 defined surge capacity as the “ability of an organization to rapidly and effectively increase [the sum of] its available resources in a specific geographic location”^12^. In 2015 an evaluation of the state of surge capacity within the humanitarian field was conducted based upon the conclusions identified in 2007^13^. Both reports discuss the gaps that continue to exist within the surge capacity of INGOs including a rising demand for surge response and the need to develop local capacity^12^^,^ 13. The findings in our study indicate that while AHGOs differ in some ways from INGOs, they have the capacity to provide surge response. This capacity is integral to AHGOs active on Lesvos, aligned with the concept of a ‘whole-organization approach’^12^. Surge capacity permeates the resolve of these AHGOs to provide aid, their commitment to assessing needs, their minimalist decision-making hierarchies and their ability to respond to changing conditions quickly. The use of untrained international volunteers of all ages, many of whom arrived on Lesvos prior to making contact, allowed AHGOs to tap into an immense volunteer pool with minimal pressure to coordinate travel arrangements.

The AHGOs responding to the crisis on Lesvos may represent a counter-example to the often-discussed Haiti earthquake response in 2010. It is widely concluded that inexperienced volunteers providing aid on the ground created new challenges for INGOs and effective disaster relief in response to the earthquake^6^^,^ 14 ^,^ 15. Following the response in Haiti, the WHO developed a classification and minimum standard for emergency medical teams and a registration process that was initially utilized during the response to Typhoon Haiyan in 2013^6^^,^ 15. Effective training of responders is considered by many to be essential to successful intervention and marks a distinction from the concept of disaster tourism^16^.

A recent challenge to the notion that professionalism is beneficial to international humanitarian relief discusses the potential negative effects of increasing distance from beneficiaries, the growth of barriers to entry within humanitarianism, and the development of organizational risk aversion^17^. Volunteer response has also not always received criticism, as reports have characterized and discussed the increasing role of online volunteer disaster support through open-source mapping initiatives and social media^18^. In response to the 2010 earthquake in Haiti, international citizen-based open source online mapping using satellite images was conducted to provide INGOs working on the ground up to date information regarding infrastructure and needs^8^^,^ 19 ^,^ 20. Inexperienced local volunteers organizations on the ground have also received acknowledgement for their positive role. Following Hurricane Sandy in October 2012, an AHGO ‘Occupy Sandy’ formed from the social network developed during the Occupy Wallstreet movement in 2011^21^. This AHGO distributed humanitarian relief to inhabitants in neighbourhoods within New York without food, water or electricity for several weeks and at its peak had approximately 60,000 local volunteers^21^.

What sets the AHGO response on Lesvos apart from previous volunteer responses is the reported relative absence of INGOs, particularly during the peak of arrivals in October 2015. This is not an absolute absence, as Medecins Sans Frontieres reports to have conducted health checks on Lesvos starting in July 2015 and other organizations may have been present, however interview respondents were harsh in their criticism of the perceived inadequate INGO and UNHCR response^22^. While inexperienced international volunteers arriving on Haiti met and jarred with the professional cadre of experienced humanitarians, inexperienced international volunteers arriving on Lesvos met only similarly inexperienced local volunteers.

A recent systematic review concluded that there was no agreed definition of international humanitarian action effectiveness characteristics, despite the global need for conducting effectiveness evaluation^23^. Through our study we have sought to characterize the AHGO disaster response on Lesvos, however, further research is required to develop evaluation frameworks for AHGO disaster response. While there are many potential issues with the AHGO response on Lesvos, such as the minimal resources to cope with post traumatic stress disorders in volunteers, utility and effectiveness of the response must be assessed before critical judgment can be made. Prior to the registration process reportedly implemented by the Greek Government, AHGOs were also able to bypass the regulatory framework developed for INGOs and work initially in a relatively empty regulatory environment. Many attempts since 1965 have been made to codify key humanitarian principles, including the International Committee of the Red Cross’ seven principles (humanity, neutrality, impartiality, independence, voluntary service, unity and universality) more recently the six core standards within the Humanitarian Charter and Minimum Standards in Humanitarian Response developed by The Sphere Project and the nine commitments advanced within the Core Humanitarian Standard^24^^,^ 25 ^,^ 26 ^,^ 27. Targeted research is needed to determine how AHGOs on Lesvos have incorporated these voluntary principles within their humanitarian action. From the results presented within this study it is clear that not all AHGOs agree with the principle of neutrality. AHGOs believed that within this disaster even positions of neutrality were inherently political.

*Future Disasters*

Diverse estimates have been made from climate modeling, ranging from tens to hundreds of millions by 2050, of newly displaced people as a result of the effects of climate change^28^. While this report outlines some of the issues with these estimates, the synthesis report for the World Humanitarian Summit indicates that it is widely expected that there may be increasing climate-induced migration within the next century^28^^,^ 29. AHGOs, as shown by those active on Lesvos, are capable evaluating needs and mobilizing resources – funding and volunteers – to respond quickly to provide humanitarian relief to those in need. While AHGOs utilize alternative methods to INGOs, they may prove effective at providing surge response in situations where INGOs have difficulty mobilizing sufficiently. Though multiple complex disasters occurring simultaneously can stretch INGO surge capacity, the experience of providing aid when INGOs are perceived to be absent echoes criticism of the international humanitarian aid system for it’s absenteeism in the field^13^^,^ 30. The unique disaster of migration on Lesvos can be considered both a drawn out crisis over many months, and a short-term crisis as new arrivals present new needs on a daily basis. AHGOs have responded to this complex disaster in the face of political pressure against the arrivals of refugees and migrants and in a field sparse with INGO and UNHCR action. These circumstances may reoccur in the future creating possible conditions that favour the development of AHGOs disaster response. Their efforts on Lesvos should be recognized and frameworks must be developed to evaluate and create favourable conditions for their existence and mobilization in future humanitarian crises.

*Limitations*

The interviews were conducted from late January to early March. During this period, there were continual changes to the environment in which these organizations worked and internal development within the organizations. The different time points of interviews may have affected the content that respondents prioritized and the elements they raised. The interviews were conducted with senior representatives from each organization, this creates the opportunity for bias in the results as senior representatives are likely to overstate the needs of the refugees and migrants, the abilities and functioning of their organizations, and the positive impact of their efforts. The interviews were conducted in English, which was a second language for some respondents. This may have limited their ability to accurately and fully describe their experiences. The accounts given are difficult to verify, as none have a complete picture of the organizations and their efforts. A strength would have been to add perspectives from refugees and migrants, volunteers, locals and other organizations active on Lesvos. However to ensure trustworthiness we have described the study population and settings, the research methods and how the analyses was made, including a team of senior international researchers, in order to improve credibility and transferability to the reader.

## Conclusion

Our study characterizes the emergent citizen humanitarian aid response present in AHGOs on Lesvos. They may prove effective at providing humanitarian aid in a surge response when INGOs are unable to respond quickly. In future complex disasters AHGOs should be recognized as new humanitarian actors and conditions should be made favourable for their operations.

## Data Availability Statement

Anonymized interview transcripts are available with DOI: 10.6084/m9.figshare.4213425.v1

## Corresponding Author

George T. Kitching: georgki@stud.ntnu.no

## Appendix A: Interview Guide Questions

1. What is the name of your organization?
2. What is the country of origin of your organization?
3. When was your organization founded?
4. Where on Lesvos is your organization active?
5. What services does your organization provide? (Follow-up question: What sort of training and services does your organization provide to your staff or volunteers?)
6. What is the total number of your staff or volunteers that are or have been active on Lesvos?
7. What was the approximate date your organization was fully operational on Lesvos?
8. What is the internal structure of your organization? (Follow-up questions: Is there a leadership or decision-making hierarchy? Are there different levels of responsibility among volunteers? How do you communicate between volunteers?)
9. Would you mind disclosing if your organization receives support from government, or from donations? (Follow-up question: How are your operations financed?)
10. What sot of coordination does your organization have wth other organizations both governmental and non-governmental on Lesvos?
11. Have your volunteers contacted you prior to arriving, or are they independent volunteers who join once they arrived on Lesvos? (Follow-up question: What sort of volunteers does your organization receive (professional background, country of origin, languages spoken)?)
12. Staff or volunteers provide humanitarian aid for different periods of time. How long are the majority of the staff or volunteers in your organization active on Lesvos? (Follow-up question: Do your staff or volunteers come back for multiple periods?)
13. What are the lessons your organization has learnt so far and how are you changing based on those lessons? (Follow-up question: What are the challenges your organization faces?)
14. Do you know of any other humanitarian aid organizations active on Lesvos who could contribute information to this study?

## References

[1] United Nations. 3RP: Regional Refugee & Resilience Plan 2015-2016. 2015.

[2] UNHCR. Press Release 4 September 2015: Statement by UN High Commissioner for Refugees, António Guterres on refugee crisis in Europe. Web Accessed on 9 May 2016 from:

[3] UNHCR. Data Report 9 May 2016: Refugees/Migrants Emergency Response ‐ Mediterranean. Web Accessed on 9 May 2016 from:

[4] European Commission. Fact Sheet: Implementing the EU-Turkey Agreement – Questions and Answers. 2016. Web Accessed on 9 May 2016 from:

[5] Wall I. The volunteer effect. IRIN news article published 10 November 2015 Web Accessed on 8 May 2016 from:

[6] World Health Organization. Classification and minimum standards for foreign medical teams in sudden onset disasters. 2013.

[7] Niamias H. Refugees in Lesbos : are there too many NGOs on the island? News article from The Guardian publishsed 5 January 2016, Web accessed on 9 May 2016 from:

[8] Harvard Humanitarian Initiative. Disaster Relief 2.0: The Future of Information Sharing in Humanitarian Emergencies. Washington, D.C. and Berkshire, UK: UN Foundation & Vodafone Foundation Technology Partnership. 2011.

[9] UNHCR. Asylum-Seekers. Web Accessed on 9 May 2016 from:

[10] UNHCR, Edwards A. “Refugee” or “migrant” - Which is right? UNHCR Viewpoint 27 August 2015. Web Accessed on 27 May 2016 from:

[11] Malterud K. Systematic text condensation: a strategy for qualitative analysis. Scand J Public Health. 2012 Dec;40(8):795-805. PubMed PMID:23221918. 10.1177/1403494812465030 23221918

[12] Houghton R, Emmens B. Surge capacity in the humanitarian relief and development sector. People In Aid. 2007.

[13] Austin L, O’Neil G. The state of surge capacity in the humanitarian sector 2015. Transform Surge Capacit Proj. 2015;1–56.

[14] Cranmer HH, Biddinger PD. Typhoon Haiyan and the professionalization of disaster response. N Engl J Med. 2014 Mar 27;370(13):1185-7. PubMed PMID:24552286. 24552286 10.1056/NEJMp1401820

[15] Brolin K, Hawajri O, von Schreeb J. Foreign Medical Teams in the Philippines after Typhoon Haiyan 2013 - Who Were They, When Did They Arrive and What Did They Do? PLoS Curr. 2015 May 5;7. PubMed PMID:26064780. 26064780 10.1371/currents.dis.0cadd59590724486bffe9a0340b3e718 PMC4447417

[16] Jacquet GA, Obi CC, Chang MP, Bayram JD. Availability and diversity of training programs for responders to international disasters and complex humanitarian emergencies. PLoS Curr. 2014 Jun 23;6. PubMed PMID:24987573. 24987573 10.1371/currents.dis.626ae97e629eccd4756f20de04a20823 PMC4073805

[17] James E. The professional humanitarian and the downsides of professionalisation. Disasters. 2016 Apr;40(2):185-206. PubMed PMID:26283645. 26283645 10.1111/disa.12140

[18] Simon T, Goldberg A, Adini B. Socializing in emergencies: A review of the use of social media in emergency situations. Int J Inf Manage. 2015;35(5):609–19. doi:10.1016/j.ijinfomgt.2015.07.001

[19] Crowley J. Connecting Grassroots and Government for Disaster Response. Washington, DC: Commons Lab of the Woodrow Wilson International Center for Scholars,. 2013.

[20] Zook M, Graham M, Shelton T, Gorman S. Volunteered information and crowdsourcing disaster relief: A case study of the Haitian earthquake. World Med Heal Policy. 2010;2(2):7–33. doi: 10.2202/1948-4682.1069 10.2202/1948-4682.1069

[21] Ambinder E, Jennings DM. The Resilient Social Network. Homeland Security Studies & Analysis Institute. 2013.

[22] Medecins Sans Frontieres. Press Release 22 March 2016 Greece : MSF ends activities inside the Lesvos “ hotspot .” Web Accessed on 8 May 2016 from:

[23] Moslehi S, Ardalan A, Waugh W, Tirone D, Akbarisari A. Characteristics of an Effective International Humanitarian Assistance : A Systematic Review. PLOS Curr Disasters. 2016;Feb 25.(1):1–15. doi: 10.1371/currents.dis.706b7dc0e8382b55a20d6f7d0cf14257. 10.1371/currents.dis.706b7dc0e8382b55a20d6f7d0cf14257 PMC4778771 26981325

[24] International Committee of the Red Cross. Proceedings of the XXth International Conference of the Red Cross. Int Rev Red Cross. 1965;(56):567–98.

[25] Bagshaw S. Humanitarian Principles. OCHA on Message. 2012.

[26] The Sphere Project. The Core Standards. Humanitarian Charter and Minimum Standards in Humanitarian Response. 2011. p. 49–78.

[27] CHS Alliance, Group URD, Sphere Project. Core Humanitarian Standard on Quality and Accountability. 2014.

[28] Brown O. The numbers game. Clim Chang Displac. 2010;8–9.

[29] World Humanitarian Summit secretariat. Restoring Humanity: Synthesis of the Consultation Process for the World Humanitarian Summit. New York, United Nations. 2015.

[30] Medecins Sans Frontieres. Where is everyone ? Responding to emergencies in the most difficult places. 2014.

